# Supplementary material for: VCAN Is Essential for ERK5-Driven Tumorigenesis in Soft Tissue Sarcoma
Source: Int J Biol Sci. 2026 Feb 4;22(5):2452–68. doi: 10.7150/ijbs.121402 (PMC12965245; doi:10.7150/ijbs.121402)
Supplement: Supplementary file 1 — Supplementary figures and tables. [file ijbsv22p2452s1.pdf]

Supp. 1

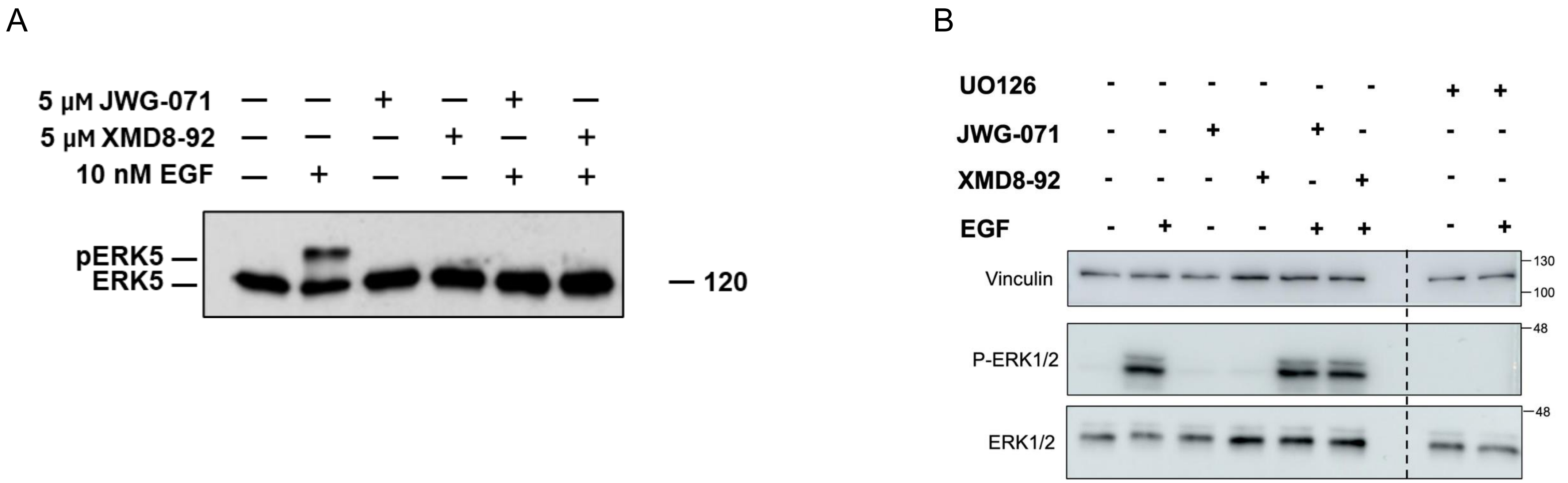

Supplementary Figure 1. Study of functionality and specificity of ERK5 inhibitors. A) SK-LMS-1 cells were starved overnight and then treated with either JWG-071 or XMD8-92 inhibitors both at 5 µM dose for 1 hour at 37ºC. Prior to lysis, cells were stimulated with 10 nM EGF for 15 minutes. Total ERK5 and pERK5 expression was determined by immunoprecipitating 1 mg of protein followed by Western Blotting with the anti-ERK5 antibody. B) SK-LMS-1 cells were starved overnight and then treated with either JWG-071, XMD8-92 or UO126 at 5 µM dose for 1 hour at 37ºC. Prior to lysis, cells were stimulated with 10 nM EGF for 15 minutes. Then 50µg of samples we blotted against indicated antibodies

Supp. Fig. 2

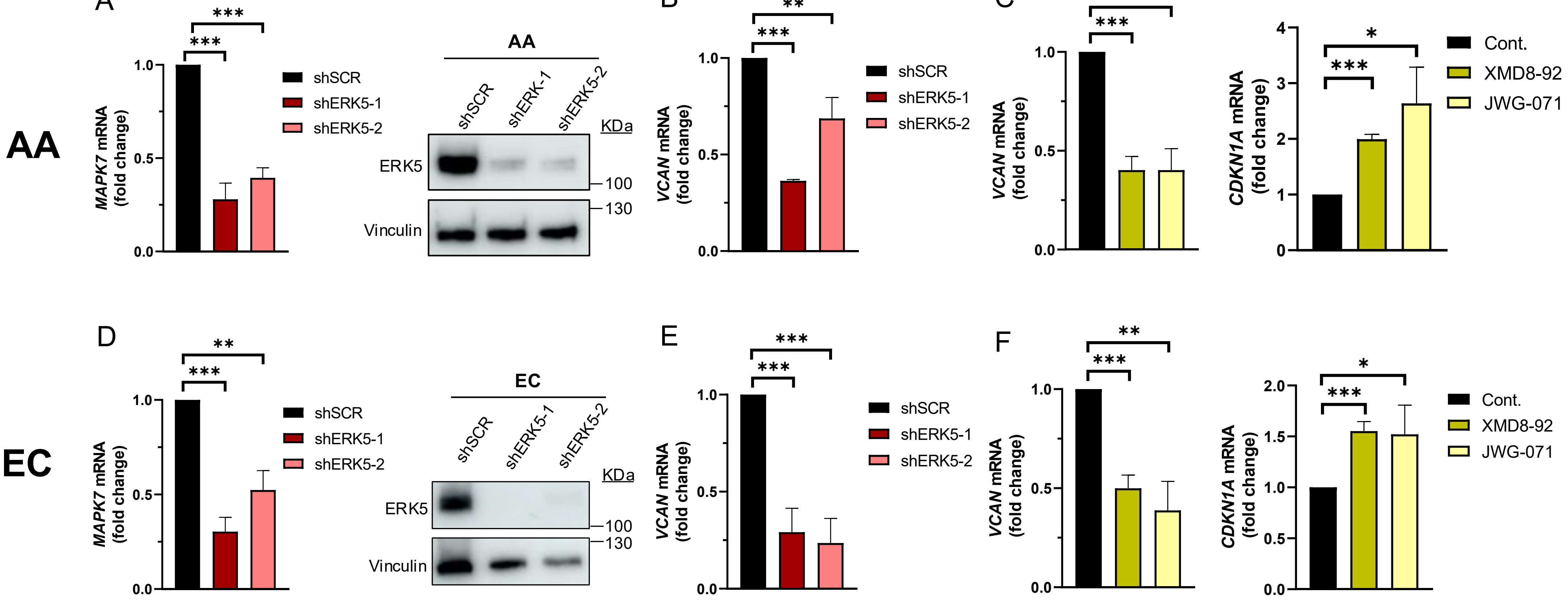

Supplementary Figure 2. ERK5 regulates VCAN mRNA levels in AA and EC cell lines. AA (A) and EC (D) cells were infected with lentiviruses carrying PLKO.1-shScramble vector (shSCR) or the human PLKO.1-shRNA ERK5 1/2 vectors (shERK5-1/2). MAPK7 mRNA levels were evaluated by RT-qPCR (left panel) and protein levels were evaluated by western blot (right panel). Vinculin was used as a loading control. VCAN mRNA levels analyzed by RT-qPCR in AA (B) and EC (E) cells infected with shSCR and shERK5-1/2 vectors. AA (C) and EC (F) cells were treated for 18 hours with the ERK5 chemical inhibitors XMD8-92 (5 µM) and JWG-071 (5 µM), and RT-qPCR was performed to analyze VCAN (left panels) and CDKN1A (right panels) mRNA levels. Graphics represent the mean +/- SD of 3 independent experiments from different pools of infection. The unpaired Student's t-test was used to assess statistical significance. **p<0.05; **p<0.01; ***p<0.001*.

Supp. Fig. 3

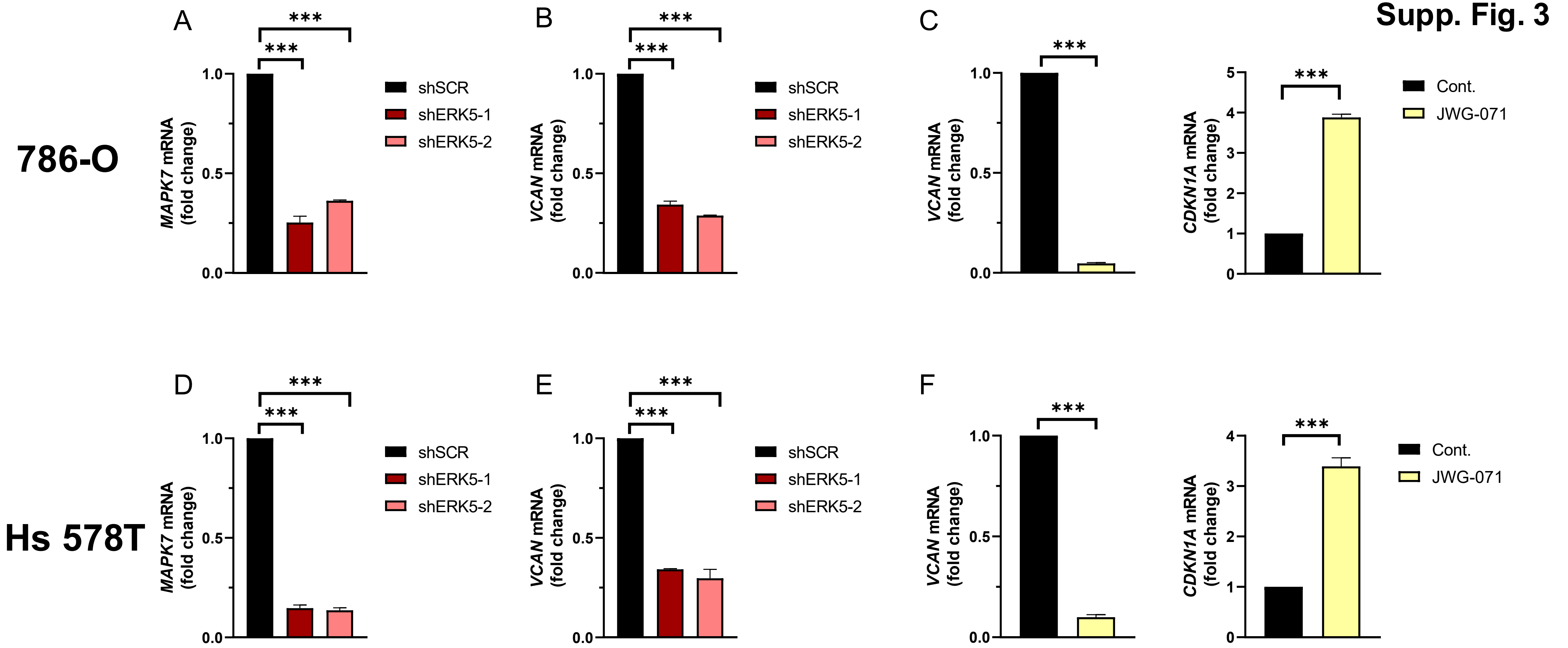

Supplementary Figure 3. ERK5 regulates VCAN mRNA levels in 786-O and Hs 578T cell lines. 786-O (A) and Hs 578T (D) cells were infected with lentiviruses carrying PLKO.1-shScramble vector (shSCR) or the human PLKO.1-shRNA ERK5 vector 1/2 (shERK5-1/2). MAPK7 mRNA levels were evaluated by RT-qPCR. VCAN mRNA levels analyzed by RT-qPCR in 786-O (B) and Hs 578T (E) cells infected with shSCR and shERK5-1/2. 786-O (C) and Hs 578T (F) cells were treated for 18 hours with the ERK5 chemical inhibitor JWG-071 (5 μM), and RT-qPCR was performed to analyze VCAN (left panels) and CDKN1A (right panels) mRNA levels. Graphics represent the mean +/- SD of 3 independent experiments from different pools of infections. The unpaired Student's t-test was used to assess statistical significance ****p<0.001*.

Supp. Fig. 4

AA

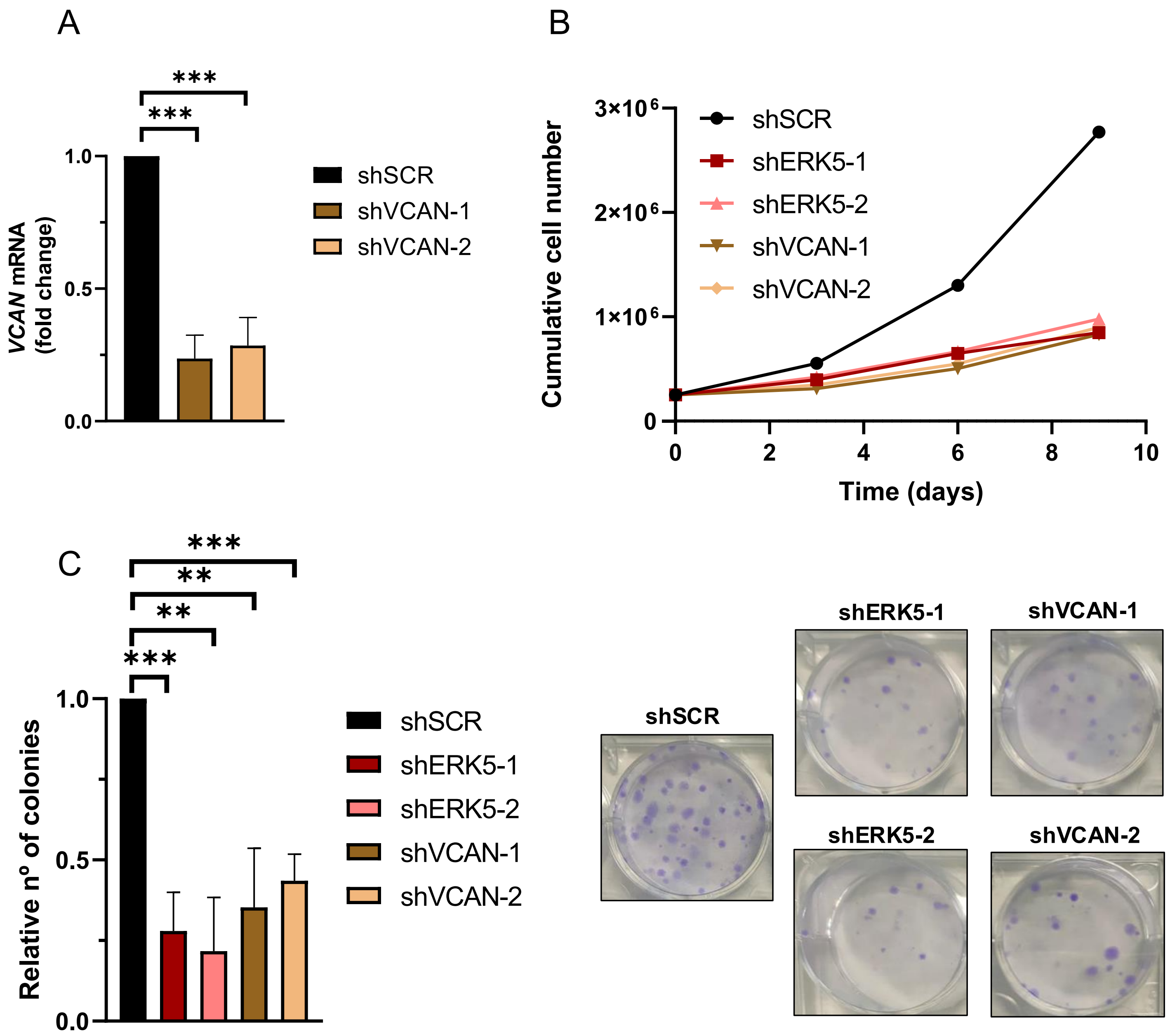

Supplementary Figure 4. ERK5 and VCAN contribute to the regulation of proliferation and colony formation capacity in AA cells. (A) AA cells were infected with lentiviruses carrying PLKO.1-shScramble vector (shSCR) or the human PLKO.1-shRNA VCAN 1/2 vectors (shVCAN-1/2). VCAN mRNA levels were evaluated by RT-qPCR. (B) For growth curves, 2.5 × 105 shSCR, shERK5-1/2 or shVCAN-1/2 AA cells were seeded in 100 mm plates. Every 3 days, cells were counted and replated in the same manner up to day 9. The graphic shows the cumulative cell number from a representative experiment out of 3 from different pools of infection with nearly identical results. (C) For evaluation of colony formation capacity, clonogenic assays were performed by seeding 400 cells/well in a 6_ well plate and they were revealed after 12-14 days. Relative number of colonies obtained in clonogenic assays of shSCR, shERK5-1/2 and shVCAN-1/2 AA cells (left panel). Representative images of clonogenic assays from the different cell lines (right panel). The graphic represents the mean +/- SD of 3 independent experiments from different pools of infections. The unpaired Student's t-test was used to assess statistical significance. **p<0.01; ***p<0.001.*

Supp. Fig. 5

EC

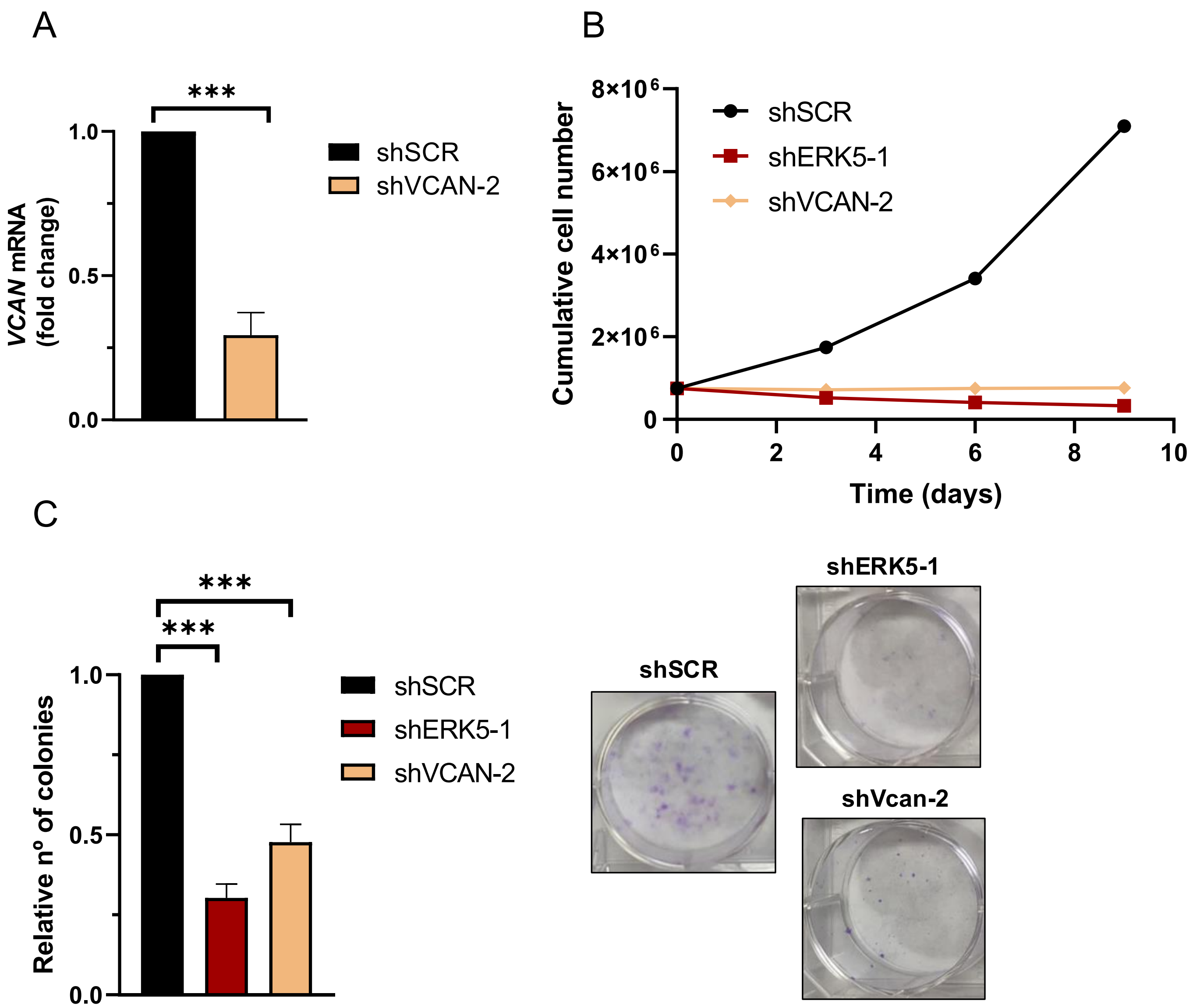

Supplementary Figure 5. ERK5 and VCAN contribute to the regulation of proliferation and colony formation capacity in EC cells. (A) EC cells were infected with lentiviruses carrying PLKO.1-shScramble (shSCR) or the human PLKO.1-shRNA VCAN-2 (shVCAN-2) vectors. VCAN mRNA levels were evaluated by RT-qPCR. (B) For growth curves, 7.5 × 105 shSCR, shERK5-1 or shVCAN-2 EC cells were seeded in 100 mm plates. Every 3 days, cells were counted and replated in the same manner up to day 9. Graphic shows the cumulative cell number from a representative experiment out of 3 from different pools of infection with nearly identical results. (C) For evaluation of colony formation capacity, clonogenic assays were performed by seeding 600 cells/well in a 6- well plate 6, and they were revealed after 12-14 days. Relative number of colonies obtained in clonogenic assays of shSCR, shERK5-1 and shVCAN-2 EC cells (left panel). Representative images of clonogenic assays from the three different cell lines (right panel). The graphic represents the mean +/- SD of 3 independent experiments from different pools of infections. The unpaired Student's t-test was used to assess statistical significance. ****p<0.001*.

Supp. Fig. 6

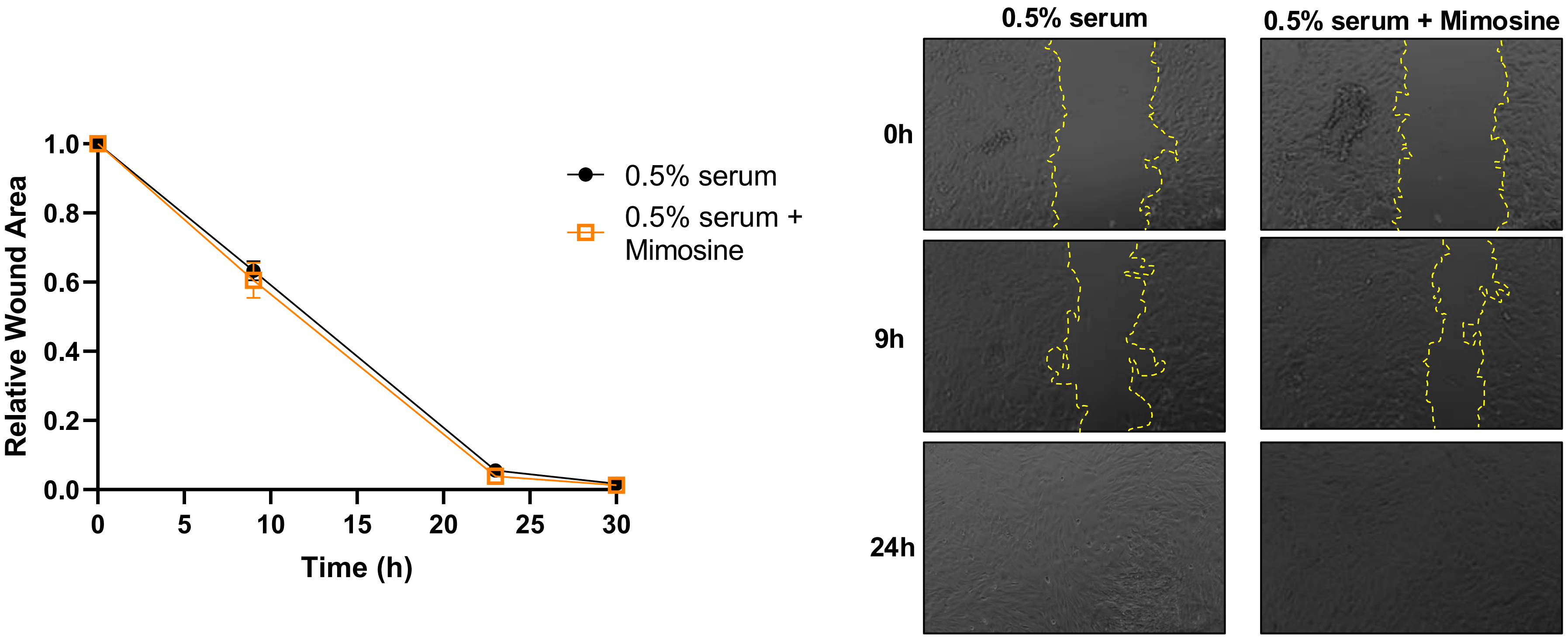

Supplementary Figure 6. Setting the conditions of wound healing assay in SK-LMS-1 cells. Relative wound area of SK-LMS-1 cells with 0,5% FBS concentration with/without mimosine treatment (400 μM) (left panel). Representative images from one out of 3 independent experiments (right panel).

Supp. Fig. 7

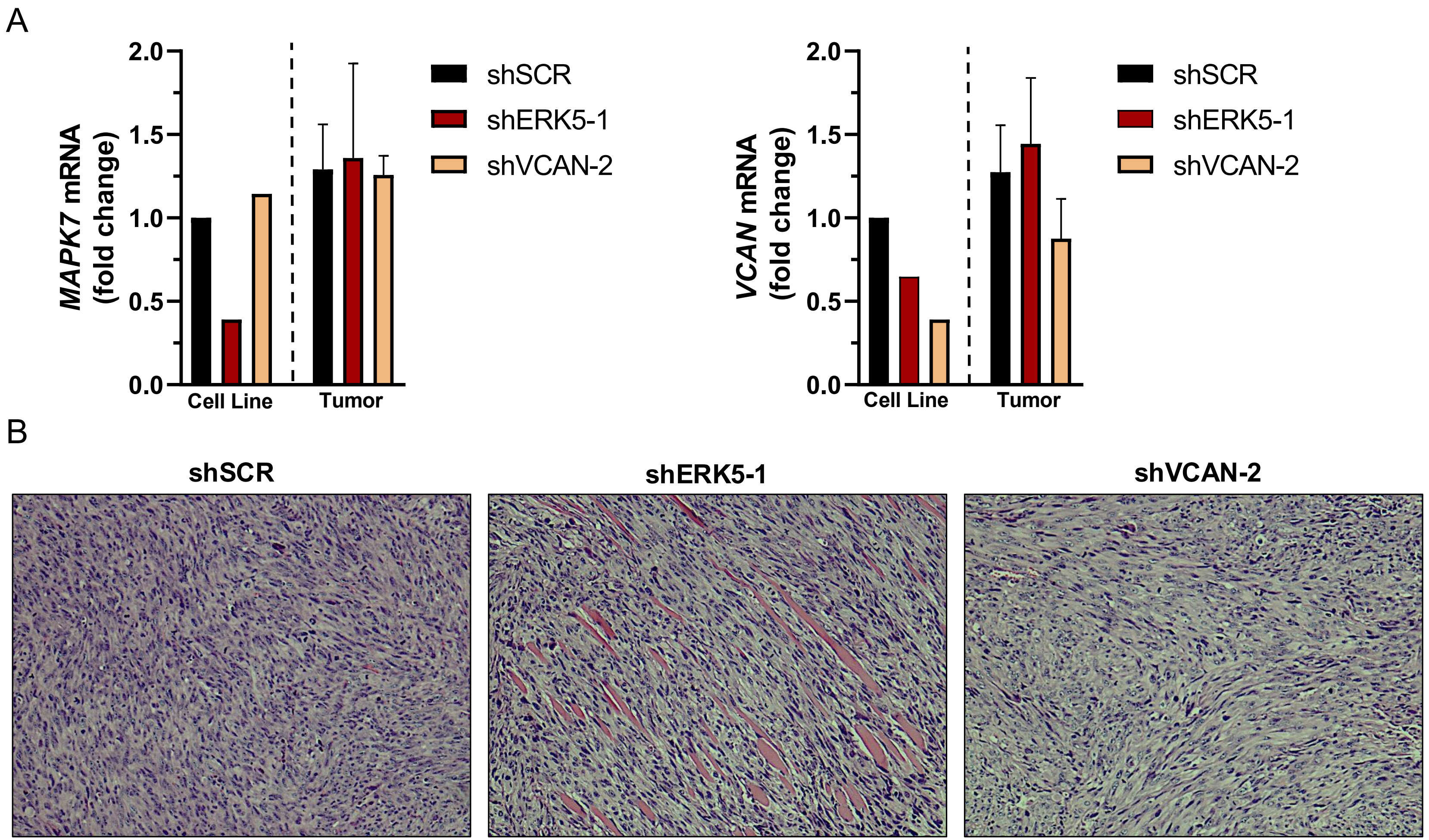

Supplementary Figure 7. Analysis of the tumors obtained in figure 4C. (A) MAPK7 (left panel) and VCAN (right panel) mRNA levels of SK-LMS-1 cells before injection (Cell Line) and from recovered tumors (Tumor), analyzed by RT-qPCR. The unpaired Student's t-test was used to assess statistical significance. (B) Representative images of hematoxylin and eosin histological study of tumors obtained from SK-LMS-1 derived cell lines, showing lesions with high cell density, spindle-shaped cells or occasionally with epithelioid appearance arranged in disorganized bundles. tumor cells also had vesicular nuclei with macronucleoli, extensive eosinophilic cytoplasm and atypical mitoses in all neoplasms. Pictures are shown at a 20X magnification.

Supp. Fig. 8

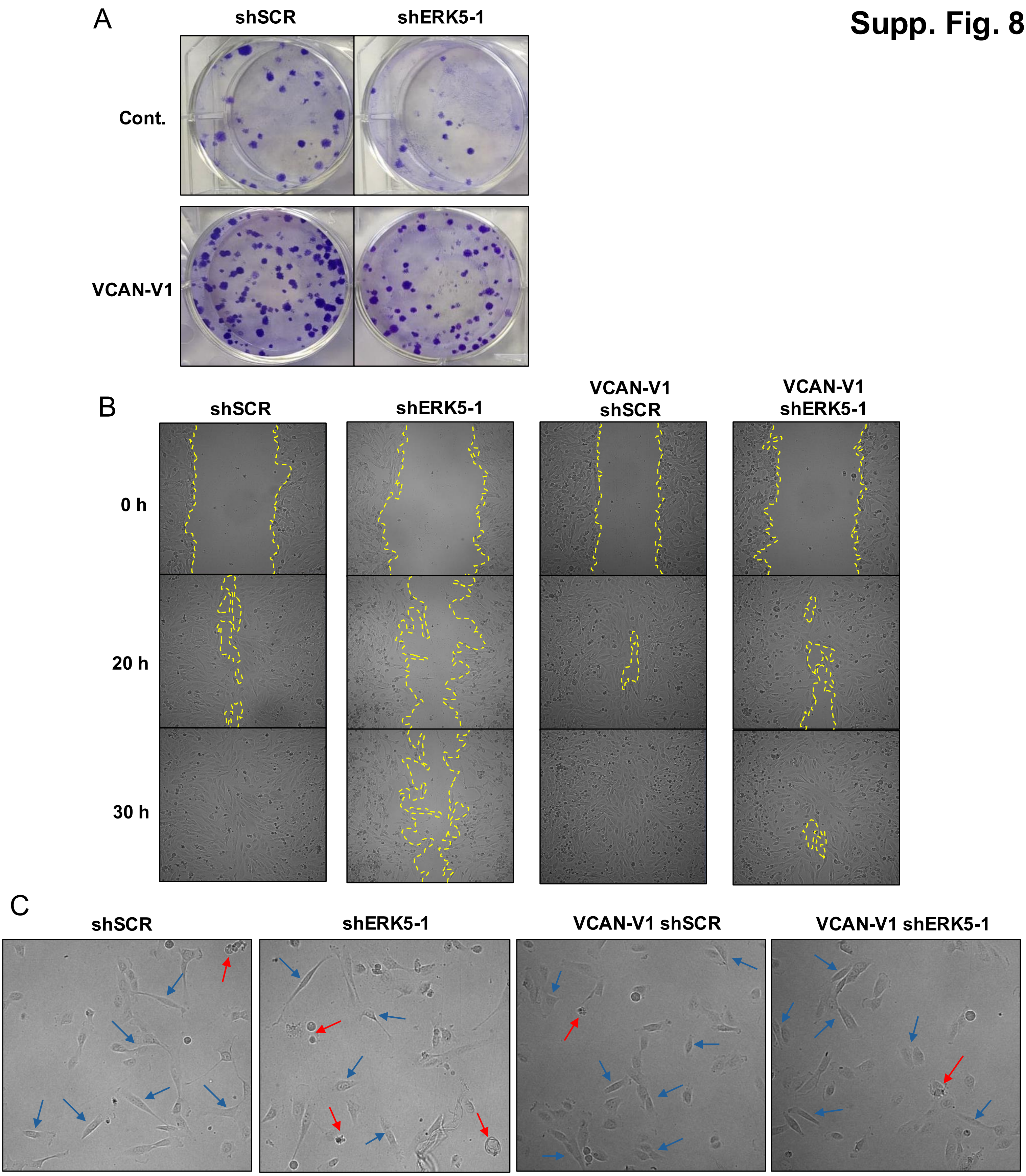

Supplementary Figure 8. Images of results presented in Figure 5. (A) Representative images of clonogenic assays from SK-LMS-1 derived cells. (B) Representative images from wound healing assays in SK-LMS-1 derived cells. (C) Representative images of SK-LMS-1 derived cells taken 300 minutes after seeding on a collagen-pretreated surface; blue arrows mark fully adherent and expanded cells; red arrows mark not attached cells

Supp. Fig. 9

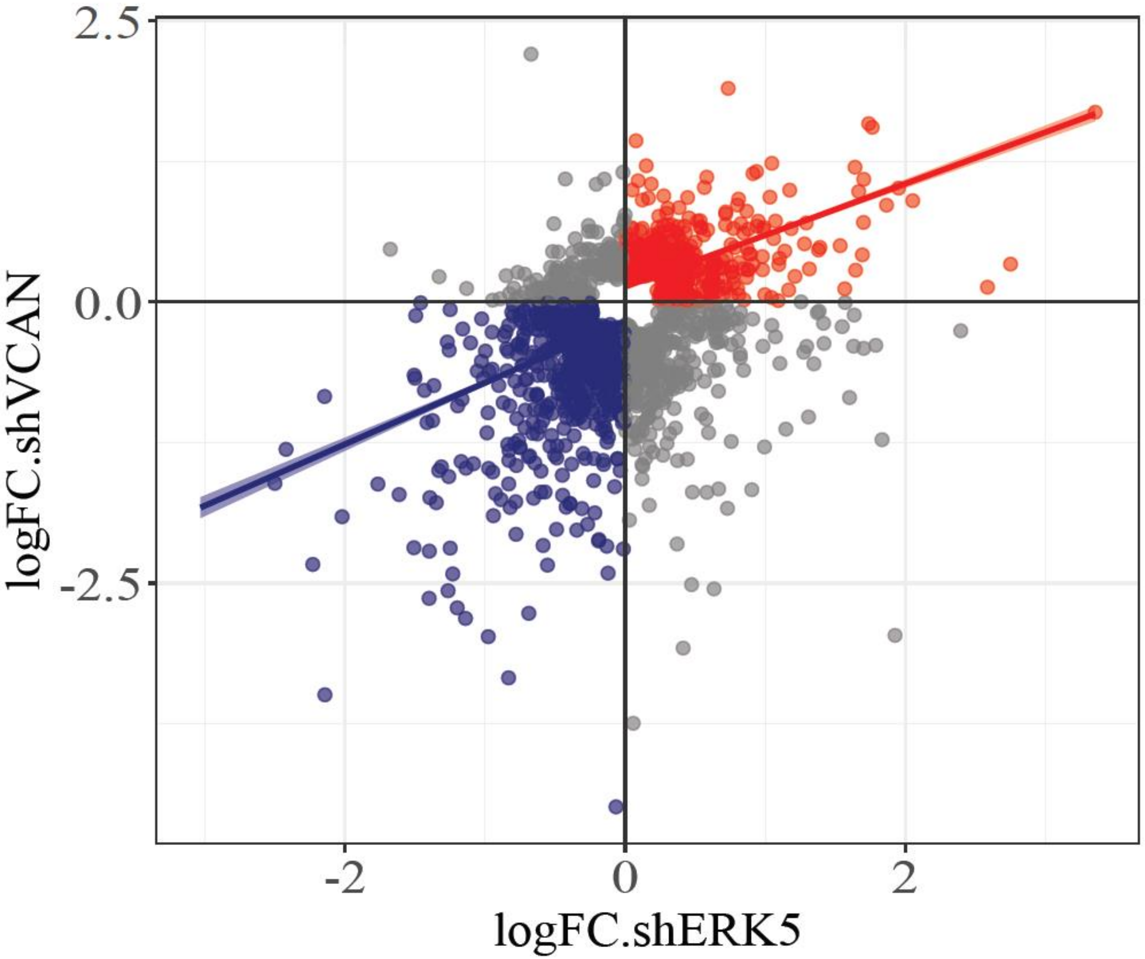

Supplementary Figure 9. Scatter plot of logFC in gene expression following VCAN versus ERK5 suppression. Each point represents a gene that was differentially expressed in at least one of the two conditions. Genes upregulated in both conditions are shown in red, those downregulated in both are shown in blue. The Pearson correlation coefficients for commonly upregulated and downregulated genes are 0.53 and 0.42, respectively ($p < 0.001$ for both).

Supp. Fig. 10

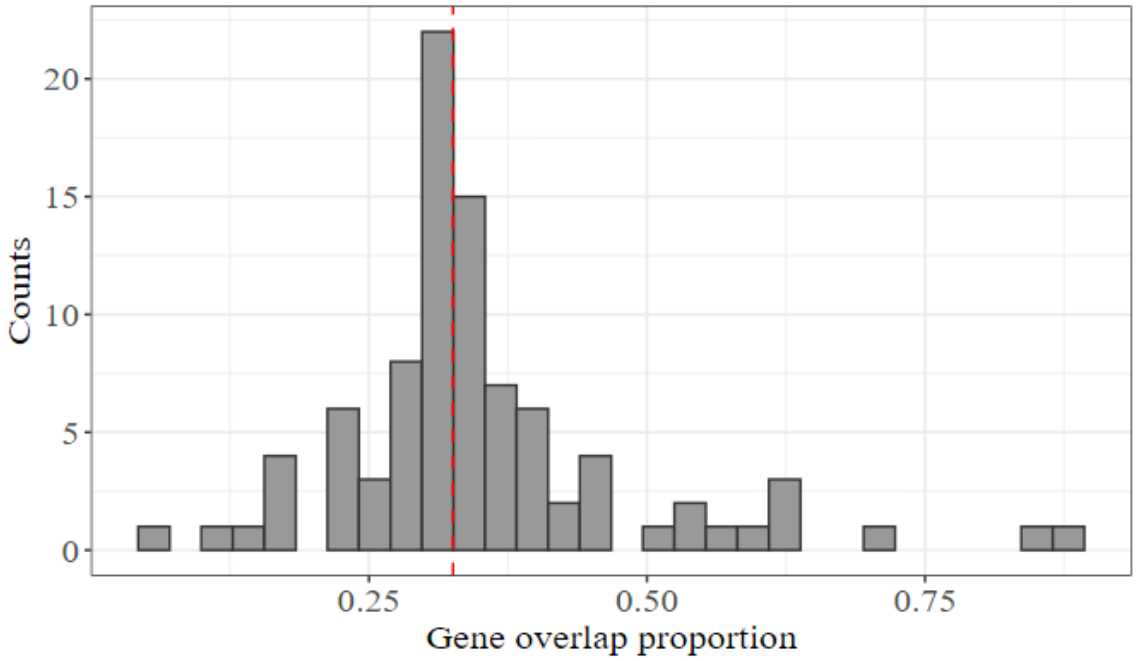

Supplementary Figure 10. Overlap of enriched biological processes between MAPK7 and VCAN interferences. Histogram shows the proportion of overlapping genes in the enriched biological processes shared between MAPK7 and VCAN interferences. For each category, the proportion is calculated by dividing the number of genes identified in the MAPK7 and VCAN deletion enrichments by the total number of genes enriched after MAPK7 deletion, with MAPK7 deletion set as the reference: $P=G_{(MAPK7 \cap VCAN)}/G_{MAPK7}$, where $G_{(MAPK7 \cap VCAN)}$ is the number of overlapping genes between MAPK7 and VCAN deletion enrichments and G_{MAPK7} is the total number of genes enriched after MAPK7 deletion. The dashed red line indicates the median overlap ratio across all categories.

Supp. Fig. 11

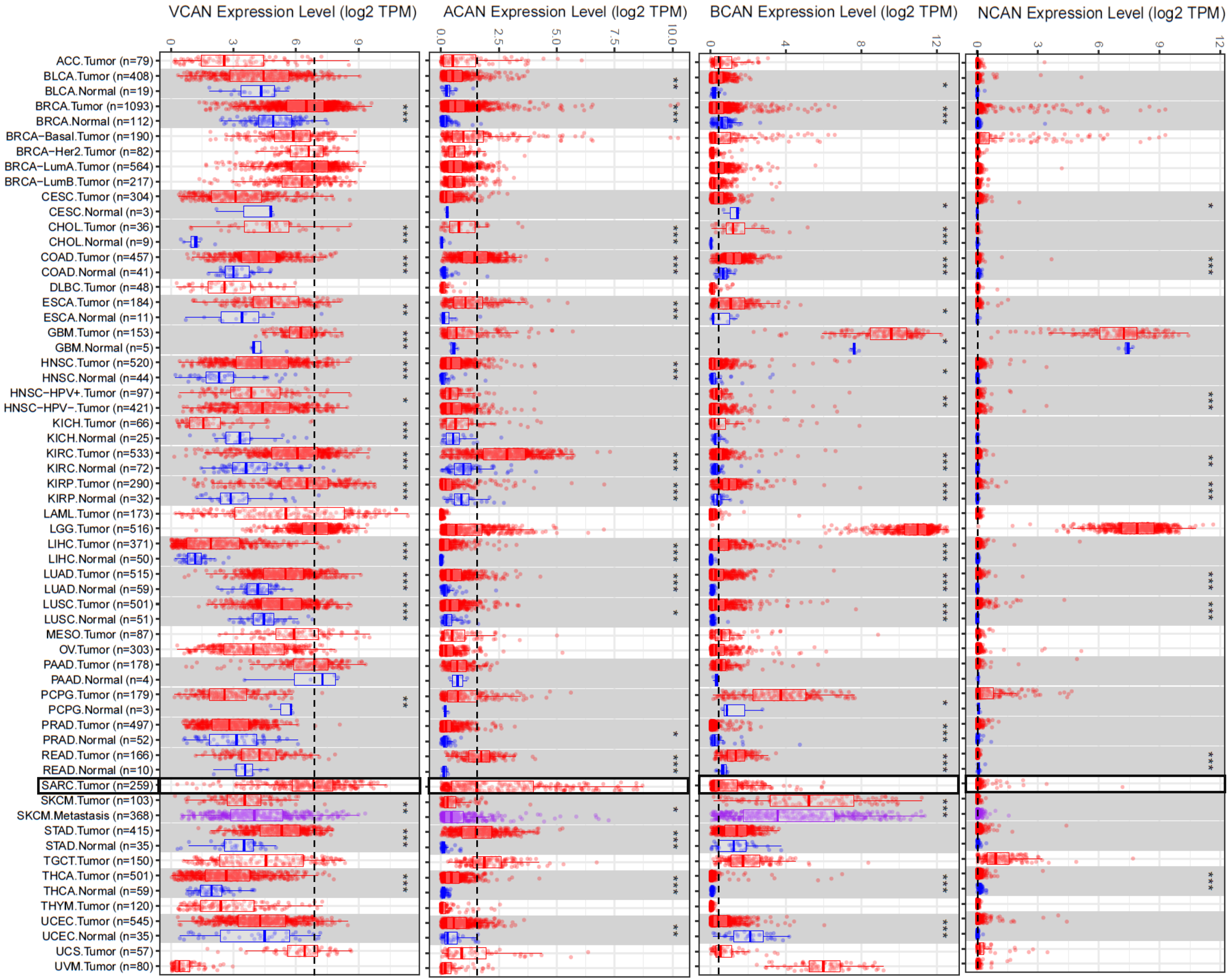

Supplementary Figure 11. mRNA expression (log2 TPM) analysis of VCAN, ACAN, BCAN and NCAN expression in samples from TCGA series using TIMER 2.0 platform. Sarcoma series (SARC Tumor) is marked with a black rectangle and the average mRNA level of each gene in sarcoma is marked with a black discontinuous line. Tumor samples depicted in red and healthy samples depicted in blue. Statistical significance of differential expression evaluated using the Wilcoxon test.

Supp. 12

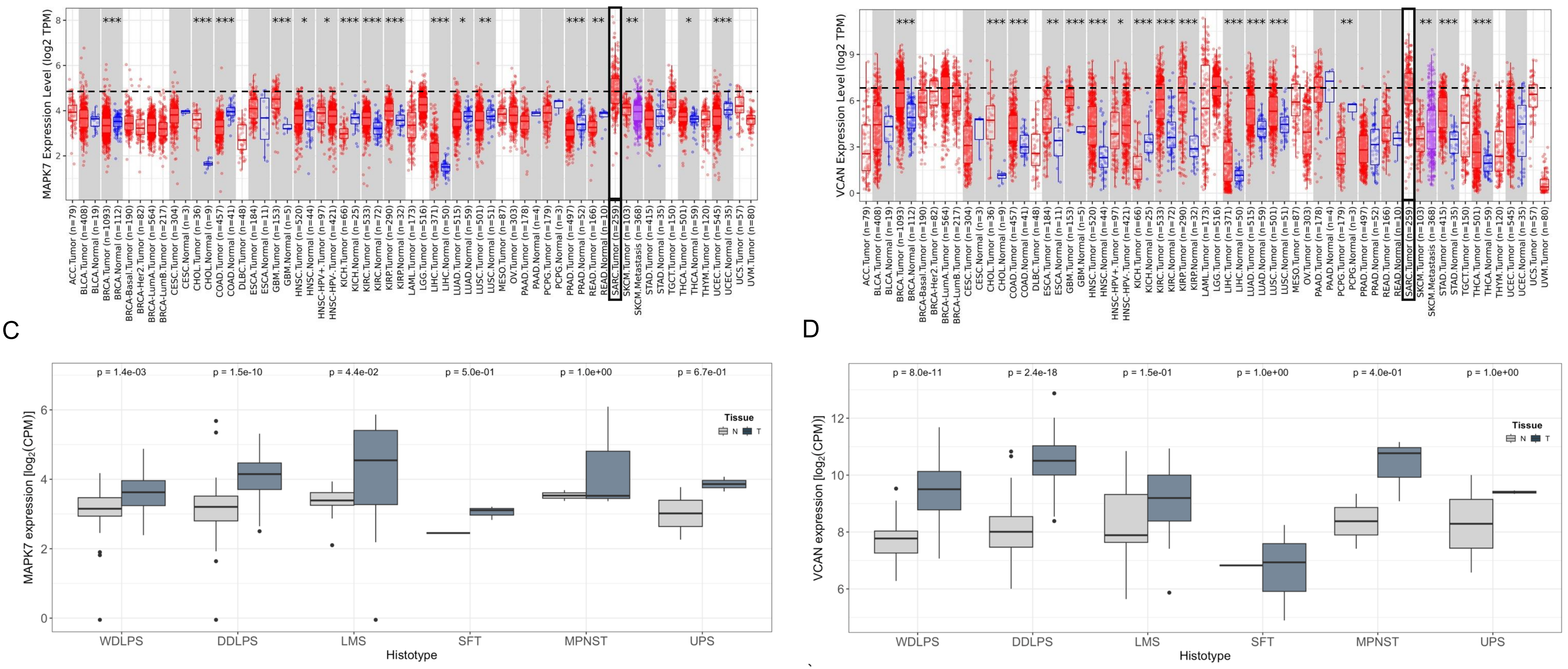

Supplementary Figure 12. mRNA expression (log2 TPM) analysis of MAPK7 (A) and VCAN (B) in the TCGA series using TIMER 2.0 platform. Soft Tissue Sarcoma series (SARC Tumor) is marked with a black rectangle and the average MAPK7 and VCAN mRNA levels in sarcoma are marked with discontinuous black lines. Comparison of MAPK7 (C) and VCAN (D) mRNA expression levels (log2 CPM) in retroperitoneal soft-tissue sarcomas (blue boxes) and paired normal tissue (grey boxes) from the SARCOMICS study by RNA-Seq. Wilcoxon test was used to address statistical significance.

WDLPS: Well-differentiated liposarcoma; DDLPS: dedifferentiated liposarcoma; LMS: leiomyosarcoma; MPNST: malignant peripheral nerve sheath tumor; UPS: undifferentiated pleomorphic sarcoma; SFT: solitary fibrous tumor.

Supp. 13

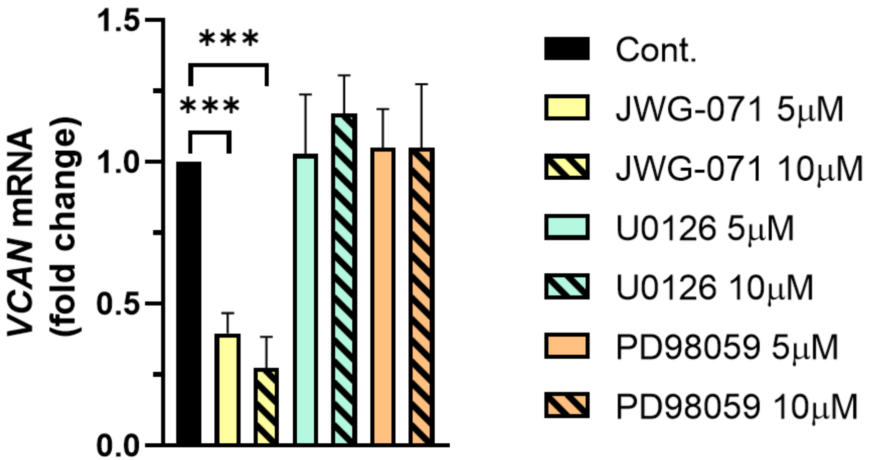

Supplementary figure 13. Effect of ERK1/2 inhibitors on VCAN expression. Subconfluent cultures of SK-LM-S1 cells were incubated with 5 and 10 μM of ERK1/2 inhibitors (U0126 and PD98059), or with the ERK5 inhibitor JWG-071 (5 and 10 μM) for 24 hours, as described in Figure 2. Samples were collected and processed for qRT-PCR to evaluate VCAN mRNA levels under the different conditions, using GAPDH as the housekeeping gene. Figure represents average of 2 independent experiments . Bars means S.D. The unpaired Student's t-test was used to assess statistical significance. *$*p<0.05$; $**p<0.01$; $***p<0.001$*.

Supplementary Table 1. List of antibodies used.

Antibody	Dilution	Manufacture	Reference	Use
ERK5	1:1000	Cell Signaling Technology	3372S	Western blot
VCAN	1:5000	Abcam	Ab19345	Western blot
MEK5	1:1000	Enzo Life Science	ADI-KAP-MA003-E	Western blot
HA	1:1000	Sigma-Aldrich	H9658	Western blot
Vinculin	1:3000	Sigma-Aldrich	V9264	Western blot
Nidogen 1	1:3000	R&D Systems	AF2570	Western blot
Calnexin	1:1000	Stressgen Bioreagents	SPC-127	Western blot
ERK1/2	1:2000	Cell Signaling Technology	4696T	Western blot
Phospho-ERK1/2	1:2000	Cell Signaling Technology	4370S	Western blot
ERK5	1μg	In house	PMID: 17317102	IP
Anti-mouse	1:3000	Cell Signaling Technology	7076S	Western blot
Anti-rabbit	1:3000	Cell Signaling Technology	7074S	Western blot
ERK5	1:500	Abcam	ab196609	Immunohistochemistry
VCAN	1:2000	Abcam	ab270445	Immunohistochemistry

Supplementary Table 2. List of primers used.

Primer	Forward	Reverse
human *MAPK7*	AGCACTTTAAACACGACAAC	TAGACAGATTTGAATTCGCC
human *VCAN*	AGGTGGTCTACTTGGGGTGA	TGGTTGTAGCCTCTTTAGGTTT
human *CDKN1A*	ACTCTCAGGGTCGAAAACGG	CTTCCTGTGGGCGGATTAGG
human *GAPDH*	TCGTGGAAGGACTCATGACCA	CAGTCTTCTGGGTGGCAGTGA
mouse *Mapk7*	AGATCTGTCTATGTGGTACTG	CTGGTACAGGAAGTATCTCAC
mouse *Vcan*	AGGCGTCTACCGATGTGATG	CAGCGGCAAAGTTCAGAGTG
mouse *B2m*	GGTCTTTCTGGTGCTTGTCTCA	GTTCGGCTTCCCATTCTCC

Supplementary Table 3. Clinical and pathological characteristics of 222 patients with either primary retroperitoneal sarcoma (RPS) or primary extremity soft tissue sarcomas (ESTS) who were included in the retrospective cohort of the SARCOMCS clinical trial and were profiled with RNA-sequencing. WDLPS: Well-differentiated liposarcoma; DDLPS: dedifferentiated liposarcoma; PLPS: pleomorphic liposarcoma; MLPS: myxoid liposarcoma; LMS: leiomyosarcoma; MPNST: malignant peripheral nerve sheath tumor; UPS: undifferentiated pleomorphic sarcoma; SS: synovial sarcoma; AS: angiosarcoma; SFT: solitary fibrous tumor; MFS: myxofibrosarcoma.

**Site**	RPS	ESTS	All
**Sex**			
Female	46 (34%)	38 (44%)	84 (38%)
Male	89 (66%)	49 (56%)	138 (62%)
**Age (median, IQR)**	63 (54.5-72)	59 (46-71.5)	62 (50-72)
**Histology**			
WDLPS	40 (30%)	0	40 (18%)
DDLPS	69 (51%)	4 (5%)	73 (33%)
PLPS	0	5 (6%)	5 (2%)
MLPS	0	14 (16%)	14 (6%)
LMS	17 (13%)	7 (8%)	24 (11%)
MPNST	3 (2%)	2 (2%)	5 (2%)
UPS	2 (1%)	23 (26%)	25 (11%)
SS	0	3 (3%)	3 (2%)
AS	0	1 (2%)	1 (1%)
SFT	4 (3%)	7 (8%)	11 (5%)
MFS	0	21 (24%)	21 (9%)
**Grading**			
I	44 (33%)	21 (24%)	65 (29%)
II	57 (42%)	20 (23%)	77 (35%)
III	34 (25%)	46 (53%)	80 (36%)
**Neoadjuvant treatment**			
Chemotherapy	7 (5%)	15 (17%)	22 (9%)
Radiotherapy	15 (11%)	9 (10%)	24 (11%)

Supplementary Table 4. Clinicopathological data from Complejo Hospitalario Universitario de Albacete sarcoma patients cohort.

ID	Age	Sex	Diagnosis	Location	ERK5 Intensity	VCAN Intensity
**Patient 01**	77	M	Leiomyosarcoma	Soft parts of the lower extremity	+++	+++
**Patient 02**	68	F	Leiomyosarcoma	Uterus	+++	++
**Patient 03**	64	F	Leiomyosarcoma	Mediastinum	0	0
**Patient 04**	44	M	Leiomyosarcoma	Rectum	+	0
**Patient 05**	58	M	Leiomyosarcoma	Axilla	0	0
**Patient 06**	48	F	Leiomyosarcoma	Uterus	0	0
**Patient 07**	49	F	Leiomyosarcoma	Lung	+	+
**Patient 08**	35	F	Leiomyosarcoma	Uterus	+	+++
**Patient 09**	58	F	Leiomyosarcoma	Uterus	+	0
**Patient 10**	56	F	Leiomyosarcoma	Uterus	+	0
**Patient 11**	55	M	Undifferentiated pleomorphic sarcom	Soft parts of the chest wall	++	++
**Patient 12**	79	F	Undifferentiated pleomorphic sarcom	Soft parts of the lower extremity	++	++
**Patient 13**	72	F	Undifferentiated pleomorphic sarcom	Meninges	0	0
**Patient 14**	71	M	Undifferentiated pleomorphic sarcom	Pelvis	+	+
**Patient 15**	70	M	Undifferentiated pleomorphic sarcom	Soft parts of the upper extremity	++	0
**Patient 16**	70	M	Undifferentiated pleomorphic sarcom	Rectum	++	++
**Patient 17**	48	F	Undifferentiated pleomorphic sarcom	Soft parts of the lower extremity	+++	+++
**Patient 18**	76	M	Undifferentiated pleomorphic sarcom	Soft parts of the lower extremity	+++	++
**Patient 19**	78	M	Undifferentiated pleomorphic sarcom	Soft parts of the neck region	++	+
